# High-Intensity Focused Ultrasound for Prostate Cancer: Long-Term Followup and Complications Rate

**DOI:** 10.1155/2012/960835

**Published:** 2012-08-15

**Authors:** Umberto Maestroni, Francesco Dinale, Roberto Minari, Paolo Salsi, Francesco Ziglioli

**Affiliations:** Department of Urology (Chief: P. Cortellini), University Hospital of Parma, Parma, Italy

## Abstract

*Introduction*. As it is well known, High Intensity Focused Ultrasound (HIFU) is a minimally invasive procedure for prostate cancer. Many investigators reported their series of patients, demonstrating the effectiveness of the treatment. The most majority of Authors, however, do not report the side effects and the complications of the procedure, which is the aim of our study. The diagnosis and management of complications is discussed, and the oncologic outcome is reported in terms of quality of life. *Materials and Methods*. We report our experience in 89 patients, low-, intermediate-, and high-risk patients according with D'Amico classification. All data collected along the study were analyzed, including side effects and complications of the procedure. *Results*. Our series demonstrates the effectiveness of the procedure, in line with larger series reported in literature by other investigators. The most important side effects are sexual function impairment and transient incontinence in a minority of cases. Minor complications are reported as well as rare cases of major complications, which can require surgical treatment.

## 1. Introduction

*Introduction*. As it is well known, High Intensity Focused Ultrasound (HIFU) is a minimally invasive procedure for prostate cancer. Many investigators reported their series of patients, demonstrating the effectiveness of the treatment. The most majority of Authors, however, do not report the side effects and the complications of the procedure, which is the aim of our study. The diagnosis and management of complications is discussed, and the oncologic outcome is reported in terms of quality of life. *Materials and Methods*. We report our experience in 89 patients, low-, intermediate- and high-risk patients according with D'Amico classification. All data collected along the study were analyzed, including side effects and complications of the procedure. *Results*. Our series demonstrates the effectiveness of the procedure, in line with larger series reported in literature by other investigators. The most important side effects are sexual function impairment and transient incontinence in a minority of cases. Minor complications are reported as well as rare cases of major complications, which can require surgical treatment.

Prostate cancer is considered one of the most important topics about male health with an important social impact on the quality of life. In Europe, it is the most common solid neoplasm with an incidence rate of 214 cases per 1,000 men [1]. The increasing life expectancy and the more and more widespread use of Prostate Specific Antigen (PSA) are probably the two most important reasons why more patients are diagnosed with prostate cancer [2, 3]. Radical surgery represents the treatment of choice in clinically localized prostate cancer and in >10 years life expectancy prostate cancer. Nevertheless, radical surgery itself can be considered a high-morbility treatment [4].

Mini-invasive procedures development, such as three-dimensional external radiotherapy, brachytherapy, or cryotherapy, especially in old or anesthesiologically high-risk patients, represents a useful treatment in prostate cancer.

HIFU (High-Intensity Focused Ultrasound) is an alternative choice in localized and low-or medium-risk prostate cancer treatment. It is a noninvasive technique inducing complete coagulative necrosis of a target tumour, without requiring surgical exposure or insertion of instruments into the lesion. 

Since April 2006 we have been treating prostate cancer with HIFU [5]; we report our experience in 100 patients and we deal with oncological outcome and secondary side effects of the procedure itself.

## 2. Materials and Methods

After obtaining local institutional approval, HIFU was introduced in our department routine. Initial training was received by an approved Ablatherm (EDAP, Lyon, France) committee. Also, our first treatments were performed under EDAP supervision. 89 patients were treated between April 2006 and December 2010. The selection criteria were cancer localized to the prostate and local relapse after radiotherapy, clinical stage, PSA, comorbidity (including anaesthetic evaluation), age over 70. Exclusion criteria were anal stenosis, previous rectal surgery, prostate size (anteroposterior diameter of the prostate cannot be longer than 25 mm due to a technical reason), and coxofemoral anchilosis. All patients were given counselling about the investigational nature of the treatment and informed consent was obtained.

We included low-, intermediate-, and high risk patients in accordance with the classification of D'Amico [6]: low risk, clinical stage T1c or T2a, Gleason score ≤6, and PSA ≤10 ng/mL; intermediate risk, PSA 10–20 ng/mL Gleason score 7 or clinical stage T2b; high risk, PSA ≥20 ng/mL, Gleason score >7, or clinical stage ≥T2b.

All patients were preliminarily unobstructed: 7 underwent Trans-Urethral Resection of Prostate (TUR-P) at the same time of the HIFU procedure; 44 underwent TUR-P two months before; others had previously unobstructed (9 underwent adenomectomy). Previous unobstruction also reduced duration of catheterization.

The characteristics of all patients are listed in Table 1. Tumours were staged using TNM staging system. None had metastatic disease. 

To perform the treatment we used a Ablatherm device (EDAP, Lyon, France): it consists of a 3.0 MHz piezoelectric therapeutic applicator and a 7,5 MHz ultrasound scanner for treatment planning. 

**Figure 1:**
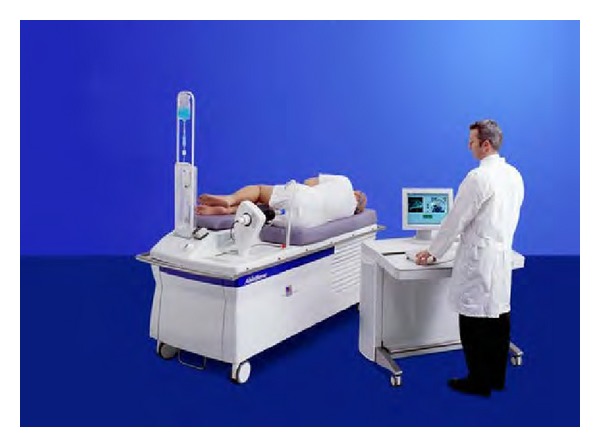
HIFU computerized surgical device.

**Figure 2:**
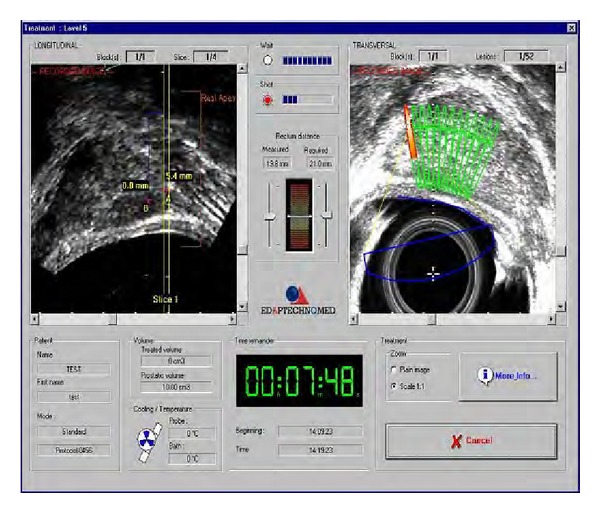
Monitor for setting and controlling the treatment procedure.

Ablatherm is a computerized surgical device (Figure 1) equipped with a treatment table, an ultrasound treatment system connected to an endorectal probe, a safety infrared ray detector, a refrigeration system keeping the rectal mucosa temperature below 14°C, and a monitor to set and control the treatment procedure through echographic screening (Figure 2).

All patients were regularly assessed based on post-HIFU PSA levels at 3, 6, 12 months and then every 6 months. Prostate biopsies (template) were performed 6 months after HIFU treatment, regardless of PSA. Prostate biopsies were also performed again during followup in cases of rise of PSA (three successive rises of PSA level).

The functional outcome was assessed using (IPSS) and (IIEF) scores: urinary symptoms and sexual potency were evaluated by IPSS—International Prostate Symptom Score, (0–7 mildly symptomatic; 8–19 moderately symptomatic; 20-35 severely symptomatic) and IIEF5—International Index of Erectile Function 5 (6–10 high erectile deficit; 11–16 moderate deficit; 17–25 low deficit; 26–30 no deficit). We collected IPSS and IIEF data before treatment and 6 months later. Incontinence data were collected from patient reported outcomes on leakage and pad usage.

Oncological failure was defined by several criteria: first of all, biochemical failure, assessed using Phoenix definition (PSA nadir + 2 ng/mL) [7]. Other criteria were starting salvage therapy, such as radiotherapy (RT) or androgen depriving therapy (ADT) and the presence of cancer on biopsy after treatment.

Data collected along experimentation was analyzed looking for risk factors. 

## 3. Results

A total of 100 HIFU procedures were performed over a 4-year period (between April 2006 and December 2011). Twenty-six patients who underwent first-line treatment were excluded because of followup <1 year as the procedure was performed in the last few months (*n* = 11), because they had their followup elsewhere (*n* = 9) or because they were not suitable for statistic evaluation, as they were not enough compliant to followup (*n* = 6). Three patients were lost to followup.

Of the remaining 74 patients, the age ranged from 65 to 80 with a mean of 72,7 years.

The proportion in the high-, intermediate-, and low-risk categories of D'Amico [6] were 13,5%, 16,2%, and 70%, respectively, with a mean (SD) PSA level of 8,07 (±8,17) ng/mL. Particularly, mean PSA level was 18,2 (±17,79) ng/mL, 10,4 (±5,04) ng/mL, and 5,8 (±2,56) ng/mL in the high-, intermediate-, and low-risk categories. Seventeen patients (28,3%) had received neoadjuvant therapy (ADT) for three months and this was discontinued immediately after HIFU.

Only seven patients underwent TUR-P at the same time of HIFU procedure.

Mean catheterization time was 9,3 days (±4,5). On the whole, 3 patients required interventions for either a stricture or endoscopic removal of necrotic tissue within the prostate cavity.

The overall mean PSA nadir was 1.12 ng/mL (±2,23), with a median of 0,95 and was obtained within a mean range of 3 ±2,3 months. A nadir value ≤0.2 was obtained in 31.6%. The nadir value was ≤1 in 76.6%.

Using the Phoenix criteria for biochemical failure, HIFU failed in 26.6% during a mean followup of 29.9 months (median 15 months, range 9–40 months).

Stratification of failure by D'Amico criteria [6] was out of the 16 failures, 43.7% high-risk, 12.5% intermediate-risk, 43.7% low-risk. In the high risk group, failures were 87.5%, in the intermediate risk group 20% and in the low risk group 16.6%. Mean time to failure was 12.5 months, with a range of 3–40 months.

During the followup, 45 patients had prostate biopsies: 15.5% were positive. All these patients had biochemical failure.

At 3 months after HIFU, 13 patients complained of urinary incontinence (see Table 1). In 6 of these patients urinary incontinence was transient and solved in 6 months. In the other 7 patients it was still present after twelve months (2 pads/die). They were investigated with urodynamic evaluation: 5 were treated with anticholinergic drugs; 2 were diagnosed with sphincteric incompetence and required artificial sphincter AMS-800.

The mean change in IPSS was 4.18 (±.4,16).

Sexual potency was defined according with the IIEF score system. 16 patients were potent before HIFU. Four men regained potency after HIFU. Four patients were partially impotent (a degree of erectile function was present but sexual intercourses were not possible) 6 months after HIFU. 5-phosphodiesterase treatment was proposed to these patients. IIEF score mean change was 11,6 (±3,6).

There was one rectovesical fistula. Diagnosis was provided by cystourethrogram and rectoscopy. This patient was managed with prolonged catheterization, as he declined any surgical procedure.

The procedure was well tolerated and no intraoperative or perioperative deaths occurred.

## 4. Discussion

Nowadays, the management of localized prostate cancer offers different approaches. Traditional established interventions, such as Radical Prostatectomy (RP) and radiation therapy (EBRT) have undergone many technical refinements in the last few years, in order to improve the clinical outcome. 

Madersbacher et al. reported the first localized prostate cancer successfully treated with HIFU in 1995 [8] and Gelet et al. published the first series in 1996 [9]. Since then, HIFU is considered as a possible alternative choice in the management of localized prostate cancer.

In 2010, Crouzet et al carried out a multicentric study on 803 patients, reporting an overall survival rate of 83% and a cancer-specific survival rate of 98% in a mean followup of 6.4 years, but more efforts are needed to gain more knowledge about side effects of the procedure and oncological outcome predictive factors [10].

In the present study, HIFU resulted in local control in 73.4% of patients, which correlates well with the results reported for the other therapeutic options. Reportedly, the risk of progression after radical prostatectomy is about 20% [11]. Radiation therapy (EBRT) results in a higher rate of recurrence. Transperineal ultrasound-guided iodine-125 brachytherapy—with or without external beam irradiation—resulted in progression in about 20% of cases [12].

To define the biochemical failure after HIFU, Phoenix definition was used (2+PSA value). There is no common agreement as to what constitutes biochemical failure after HIFU. Different definitions have been proposed and used by other investigators for biochemical failure, such as Stuttgart definition [13]. However, in the largest reports to date of long-term oncological outcome after HIFU, Phoenix definition is used [10, 14].

In the present study, prostatic biopsy was also performed, as the use of combined criteria is certainly the best for evaluating the efficacy of HIFU treatment.

Our data show the oncological outcome of 74 patients after HIFU, with a mean followup of 29.9 months. As it is clearly reported in Table 1, the highest rate of biochemical failure was found in the high-risk group (87.5%), while the lowest rate was found in the low-risk group. The high rate of failure found in the high-risk group is also due to the small number of high-risk patients treated with HIFU. The most favourable outcome is reported in low- and intermediate-risk group. This correlates well with the results reported by many investigators.

The most common side effects of HIFU for prostate cancer include prolonged voiding dysfunction and retention caused by edema, necrosis, or bladder outlet obstruction. Combination therapy (TUR-P+HIFU) reduces these side-effects, thus improving the quality of life in the postoperative time [15].

The rate of adverse events is low (see Table 1). Grade I stress incontinence was observed in 5% to 11% of patients, grade II in up to 4% patients, and grade III incontinence is rare. Rectovesical fistula is a rare event, also. In our series only one case of fistula is reported.

The main consequence of HIFU treatment on quality of life is erectile function impairment. In this field, our results are in line with literature. Preservation if erectile function is dependent on the position of the tumoral lesion. Even if sparing the contralateral side for bundle preservation can improve potency, as reported by Poissonnier et al. [16], this results in a higher failure rate [17]. For this reason, in our series sparing technique was not performed.

The major complication of this treatment is rectovesical fistula, as reported in one case in our series. The addiction of the cooling system has dramatically decreased the incidence of this complication, which reportedly ranges between 0.5–1,2% [18].

In the most majority of cases, this complication can be managed with a conservative treatment, such as long-term catheterization. In selected cases major surgery is required. 

Many investigators have confirmed the efficacy and effectiveness of HIFU treatment, but definitive data are not yet available, due to short followup and different definition of end points (by biochemical, disease-free survival rates), thus leading to difficulties and misunderstanding in results interpretation. Also, European guidelines for prostate cancer do not define a precise indication for HIFU treatment, which is still considered an alternative therapeutic option in patients diagnosed with localized prostate cancer. Our data contribute to demonstrate the positive oncological outcome in a four-year followup and to define the incidence of the most common complications. 

## Figures and Tables

**Table 1:** Complications and complication rate after HIFU treatment.

Complications	Patients	Patients rate
Grade I stress incontinence	4–7	5 to 11%
Grade II stress incontinence	3	4%
Grade III stress incontinence	0	—
Dysuria	7	10%
Impotence	67	90%
Bladder outlet obstruction	3	4%
Fistula	1	—
